# Dislocations and deformation microstructure in a B2-ordered Al_28_Co_20_Cr_11_Fe_15_Ni_26_ high-entropy alloy

**DOI:** 10.1038/srep29700

**Published:** 2016-07-19

**Authors:** Michael Feuerbacher

**Affiliations:** 1 Peter Grünberg Institut PGI-5, Forschungszentrum Jülich GmbH, D-52425 Jülich, Germany

## Abstract

High-entropy alloys are multicomponent metallic materials currently attracting high research interest. They display a unique combination of chemical disorder and crystalline long-range order, and due to their attractive properties are promising candidates for technological application. Many high-entropy alloys possess surprisingly high strength, occasionally in combination with high ductility and low density. The mechanisms effecting these attractive mechanical properties are not understood. This study addresses the deformation mechanism of a Al_28_Co_20_Cr_11_Fe_15_Ni_26_ high-entropy alloy, which is a two-phase material, consisting of a B2-ordered matrix and disordered body-centred inclusions. We quantitatively analyse the microstructure and dislocations in deformed samples by transmission-electron-microscopic methods including weak-beam imaging and convergent-beam electron diffraction. We find that the deformation process in the B2 phase is dominated by heterogeneous slip of 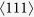 screw dislocations gliding on 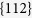 planes. The dislocations are perfect superdislocations of the B2 lattice and show no dissociation. This indicates that the antiphase-boundary energy in the structure is very high, inhibiting spread of the dislocation core. Along with the observation of a widely extending strain field associated to the dislocations, our results provide a possible explanation for the high strength of this high-entropy alloy as a direct consequence of its dislocation structure.

High-entropy alloys (HEAs) are solid solutions typically containing five or more elements of equiatomic or near-equiatomic composition. In the ideal case HEAs solidify as a single phase of random chemical order on a simple crystal lattice^1^,^2^. To date, the existence of HEAs in several alloy systems, of body-centred cubic (bcc) and face-centred cubic (fcc)^3^ as well as hexagonal^4^ structure has been reported. Besides these ideal HEAs, several systems with more than one phase, but still fewer phases than the maximum allowed according to Gibbs’ phase rule, are known^5^. Typically these materials (occasionally referred to as compositionally complex alloys) contain two or three phases.

The simultaneous presence of topological order and chemical disorder involving numerous elements is unique in metals. It discriminates HEAs from metallic glasses and conventional crystals and poses fundamental questions in basic science. HEAs also display a variety of technologically appealing and tuneable properties, such as high hardness, strength and ductility, oxidation and wear resistance, magnetism, etc., which render them attractive for application (e.g. ref. 6). HEAs also show attractive, in conventional materials mutually excluding properties, such as, for example, low density *and* high hardness^7^.

Several HEAs display exceptionally high strength in tensile and compression testing. Yield stresses of about 2.5 GPa^8^,^9^ and fracture stresses approaching 4 GPa^10^ at room temperature have been reported in alloys containing more than one phase. Single-phase bcc alloys can also be remarkably strong: the refractory single-phase HEA VNbMoTaW shows a 0.2% yield stress of 1246 MPa at room temperature^11^. Single phase FeCoCrMnNi was demonstrated to show fracture-toughness values above 200 MPa m^1/2^ 12, which exceeds the toughness of virtually all pure metals and alloys. Al-rich HEAs in the Al-Co-Cr-Fe-Ni alloy system with a B2 ordered matrix structure, the topic of the present report, are among the strong HEAs, showing 0.2% yield stresses of about 1250 MPa at room temperature^13^. Multiphase Al-Co-Cr-Cu-Fe-Ni HEAs were also demonstrated to behave superplastically, with an elongation capability up to 860% of plastic strain^14^.

In the literature it is often argued that the high strength of HEAs is a direct consequence of a solid-solution strengthening effect in an extreme form due to the high concentration of solute atoms in the HEAs^1^,^15^,^16^. This concept is questionable^17^ for several reasons: First, conventional solid-solution hardening theories^18^ cannot be extrapolated to the concentration ranges encountered in HEAs, since the concentration limits assumed in their derivation typically are by far exceeded. Second, in an HEA is not *a priori* clear which atom species are to be considered solute and which native. Third, the explanation is too general in the sense that there also exist HEAs, for example in single phase fcc systems^19^,^20^, that are not particularly strong, showing 0.2% yield stress values of about 200 MPa at room temperature. Fourth, the solid solution-strengthening hypothesis is occasionally made (e.g. ref. 16) without taking into account that, particularly in multi-phase systems, other strengthening mechanisms such as e.g. particle hardening may also operate.

Whatever the mechanism leading to the high strength of HEAs is, its identification and understanding requires thorough microstructural investigation of deformed HEAs and analysis of the dislocations involved in the process. Few dedicated studies are available in the literature to date. Otto *et al*. characterized dislocations in deformed fcc FeCoCrMnNi HEAs^21^, and dislocations and deformation properties of bcc HEA phases in the refractory system Zr-Nb-Ti-Ta-Hf were investigated by Couzinié *et al*.^22^ and Feuerbacher *et al*.^17^. No work on dislocation analysis in the system Al-Co-Cr-Fe-Ni has been published yet.

In this study we investigate the microstructure of a plastically deformed Al_28_Co_20_Cr_11_Fe_15_Ni_26_ HEA. The HEAs towards the Al-rich region of the Al-Co-Cr-Fe-Ni system, including the equiatomic composition, consist of two phases, a B2 ordered matrix with disordered, bcc inclusions, the volume proportions of which change with composition. The investigated alloy has a high matrix proportion, which allows for a detailed and quantitative analysis of the dislocations mediating the deformation process. For the first time in an HEA, we employ weak-beam imaging and convergent-beam electron diffraction techniques for a complete and unambiguous determination of dislocation Burgers vectors. We determine the slip geometry and relate the results to comparable intermetallic compounds.

## Methods

An ingot of composition Al_28_Co_20_Cr_11_Fe_15_Ni_26_ was produced using high-purity raw materials in a high-frequency levitation furnace. The ingot was used as a melt for Czochralski growth under 650 mbar argon atmosphere using a heterogeneous tungsten seed at a pulling rate of 1 mm/h. The Czochralski process is a near-equilibrium growth method and involves annealing of the grown crystal for several hours. Therefore the material produced can be considered well equilibrated.

The resulting crystal consisted of several grains, with an average grain size of about 1 mm^3^. It was characterized by scanning electron microscopy using a JEOL 840 instrument equipped with EDAX energy dispersive X-ray analysis and scanning transmission electron microscopy (STEM) using a FEI Titan 80–300 equipped with a high-angle annular dark-field detector. The crystal grown consists of an Al-Ni rich matrix with spherical inclusions of a density of about 2 vol.% (see Supplementary information Fig. S1a). The matrix was identified by high-resolution STEM and electron diffraction as a B2 structure^23^ (see Supplementary information Figs S1b and S2). The spherical inclusions are rich in Cr and Fe and of disordered bcc structure. Their size distribution is highly monodisperse, the diameter of most particles amounting to about 17 nm. Their lattice is perfectly congruent with the matrix (see Supplementary information Fig. S1b). This was also observed for equiatomic AlCoCrFeNi HEAs^24^, which is constituted by the same structures at a lower matrix proportion^25^. A detailed characterization and description of the microstructure will be provided in a forthcoming publication.

Compression tests were carried out at room temperature at constant strain rate of 10^−4^ s^−1^ in a modified Zwick Z050 uniaxial tensile testing machine on cuboidal samples of about 2 × 2 × 5 mm^3^. The sample investigated was deformed up to a plastic strain of 0.5% and then rapidly unloaded. The stress-strain curve is shown as Supplementary information Fig. S3.

Specimens for transmission electron microscopy (TEM) were prepared by subsequent grinding and argon ion milling. Dislocation analysis was carried out by two-beam Bragg-contrast imaging and convergent-beam electron diffraction (CBED) using a Philips CM20 transmission electron microscope.

## Results

**Figure 1:**
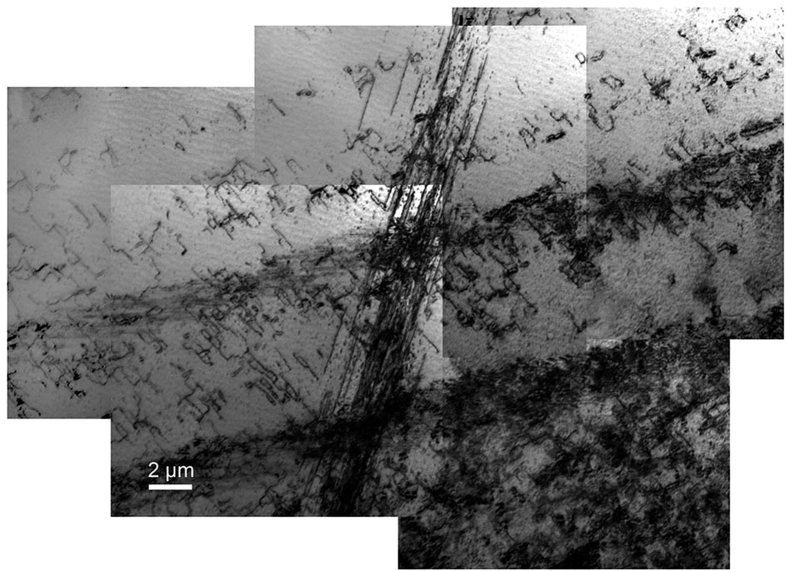
Bright-field Bragg-contrast image displaying the typical microstructure of the deformed Al_28_Co_20_Cr_11_Fe_15_Ni_26_ high-entropy alloy. The image is composed of five overlapping single TEM micrographs. Imaged using the reflection (−1 0 1) at the [1 0 1] zone axis.

Figure 1 is a bright-field Bragg-contrast image of the deformed sample and represents its typical microstructure. It is composed of five overlapping single TEM micrographs, taken using the reflection (−1 0 1) at the [1 0 1] zone axis. The sample contains dislocations, imaged as dark lines on a grey background, which are mostly located in slip bands of differing density. The density of the slip bands in Fig. 1 distinctly increases from the upper to the lower part of the image. Slip bands are present in different orientations. In Fig. 1, the group of parallel, almost horizontal bands is crossed by a rather vertical single band in the centre.

**Figure 2:**
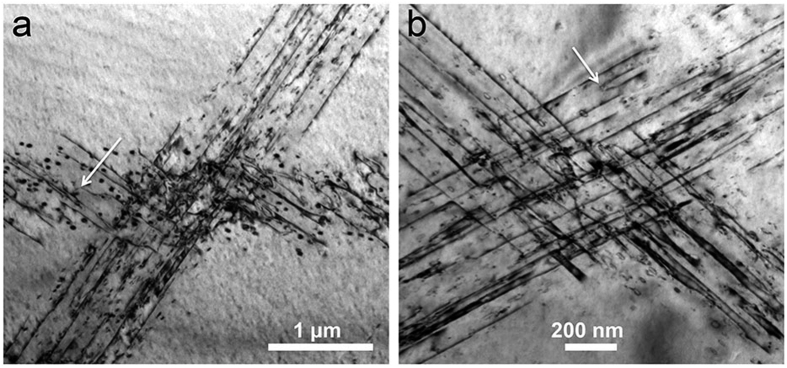
Bright-field Bragg-contrast images of the crossing of two slip bands in the deformed Al_28_Co_20_Cr_11_Fe_15_Ni_26_ high-entropy alloy. (**a**) Imaged using the (0 0 2) reflection close to the [1 0 0] zone axis. (**b**) Imaged at slightly larger magnification using the (−1 0 1) reflection close to the [1 0 1] zone axis.

Figure 2 displays Bragg-contrast images of the crossing of two slip bands imaged at different zone axes. Figure 2a is imaged using the (0 0 2) reflection close to the [1 0 0] zone axis. Both bands contain straight and parallel dislocation segments as well as small dislocation loops of about 30 to 50 nm in diameter. While in the diagonal band the straight segments are oriented parallel to the direction of the band, they lie at an angle of about 30 degrees in the horizontal band. Figure 2b is a slightly higher magnified micrograph of the same crossing imaged using the (−1 0 1) reflection close to the [1 0 1] zone axis. At this imaging orientation, the straight dislocation segments in both bands are at an angle with respect to the orientation of the band. The central area contains tangles, indicating dislocation interaction at the crossing. Note that at some of the dislocations a short, faint fringe contrast extending from the dislocation line can be seen (e.g. in the upper left corner of Fig. 2b).

**Figure 3:**
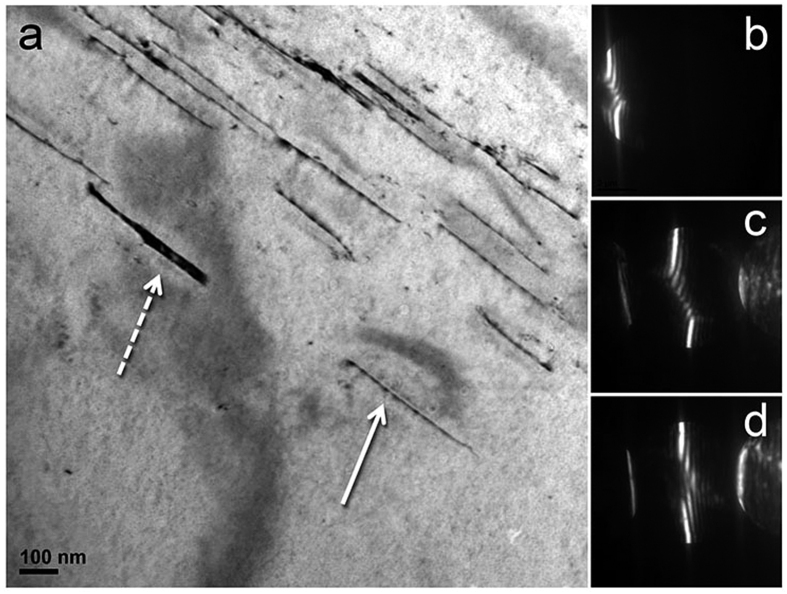
Analysis of dislocations in the deformed Al_28_Co_20_Cr_11_Fe_15_Ni_26_ high-entropy alloy. (**a**) Bright-field Bragg-contrast micrograph imaged using the (−1 0 1) reflection close to the [1 0 1] zone axis. (**b**,**c**) CBED pattern of the single dislocation (solid arrow). (**d**) CBED pattern of the narrow pair of dislocations (dashed arrow).

Figure 3a is a Bragg contrast micrograph, imaged using the (−1 0 1) reflection at the [1 0 1] zone 4axis. It displays a less dense band containing mainly straight, single dislocations, (e.g. marked by the solid arrow) and a narrow pair of dislocations (dashed arrow). Again, the straight single dislocations are connected to a faint local fringe contrast. Imaging under two-beam conditions using different reflections reveals that the contrast of the dislocations in the slip band is extinct using the (0 1 −1) reflection at the [1 0 0] zone axis and using the (1 2 −1) reflection at the [1 0 1] zone. Hence, we can conclude^26^ that the Burgers vector is parallel to the [−1 1 1] direction. In order to determine the length of the Burgers vector we have analysed the dislocation marked by a solid arrow by CBED. The (0 0 −4) Kikuchi band at the [1 0 0] zone displays fourfold splitting (Fig. 3b). The Cherns-Prestion condition, relating the reciprocal lattice vector **g** associated to the Kikuchi band and the Burgers vector **b** to the number of splittings thus reads as **g·b** = −4. The sign of the right-hand side was determined following the procedure described by Feng and Wang^27^. The (−3 0 3) Kikuchi band at the [1 0 1] zone shows sixfold splitting (Fig. 3b) and fulfils the relation **g·b** = 6. With these results and taking the contrast extinctions into account, we can determine the Burgers vector of the dislocation as **b** = a [−1 1 1], where a is the lattice parameter. The Burgers vector is hence a full body diagonal of the cubic unit cell, and with a = 0.29 nm^23^, its length amounts to 0.50 nm. Fig. 3c is a CBED pattern using the (−3 0 3) Kikuchi band at the [1 0 1] zone of the very narrow pair of dislocations marked by the dashed arrow. The CBED pattern reveals that a strain field locally exists and leads to splitting of the Kikuchi line, but the total Burgers vector of the pair is zero.

We have carried out Burgers-vector determinations following this procedure for numerous straight dislocations in several slip bands in about 20 different locations and in different specimens taken from the deformed sample. We consistently and exclusively find Burgers vectors of type a 〈1 1 1〉, where a is the lattice parameter. In a given slip band we find that all straight and parallel dislocations have identical Burgers vector. Burgers vectors of both signs are present, but in a given band one direction always is predominant.

In order to determine the dislocation line direction in space we have carried out tilting experiments, imaging straight dislocations along different zone axes and analysing the projected line. Using images along the [1 0 0], [1 0 1] and [3 –1 1] zone axes, we find that the line direction of the straight dislocations in Fig. 3a is parallel to [−1 1 1]. These dislocations are hence pure screw dislocations. Generally we find, analysing diverse examples at various specimen locations, that all straight dislocations are screws. Occasionally edge segments can also be found, see e.g. the arrows in Fig. 2. These, however, are always much shorter than the screw segments.

**Figure 4:**
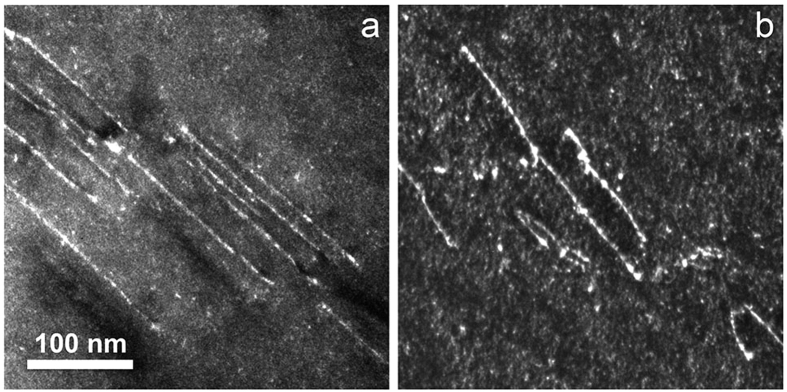
Weak-beam dark-field image of dislocations in a slip band. (**a**) Using the (−3 0 3) reflection at the [1 0 1] zone axis. (**b**) Using the (0 0 −4) reflection at the [1 0 0] zone (**b**).

Dark-field and weak-beam imaging of the screw dislocations did not reveal any indication of dislocation dissociation. Figure 4a is a weak-beam dark-field image of a slip band using the (−3 0 3) reflection at the [1 0 1] zone axis, Fig 4b is imaged using (0 0 −4) at the [1 0 0] zone. No dissociation of the dislocations is resolved. We obtained the same result at various relative orientations of the incident electron beam and the slip plane, and conclude that a dissociation, if present, has a width of well below 1 nm.

If in a micrograph the straight dislocation segments are aligned parallel to the slip band, the latter is in an edge-on orientation, and we can hence determine the slip-plane normal. This is e.g. the case for the diagonal band in Fig. 2a, for which we find a (−1 2 1) slip plane. Generally we find slip planes of type {1 1 2}. The edge-on orientation of the slip band also allows for the determination of its thickness, which amounts to about 1 μm in Fig. 2a.

**Figure 5:**
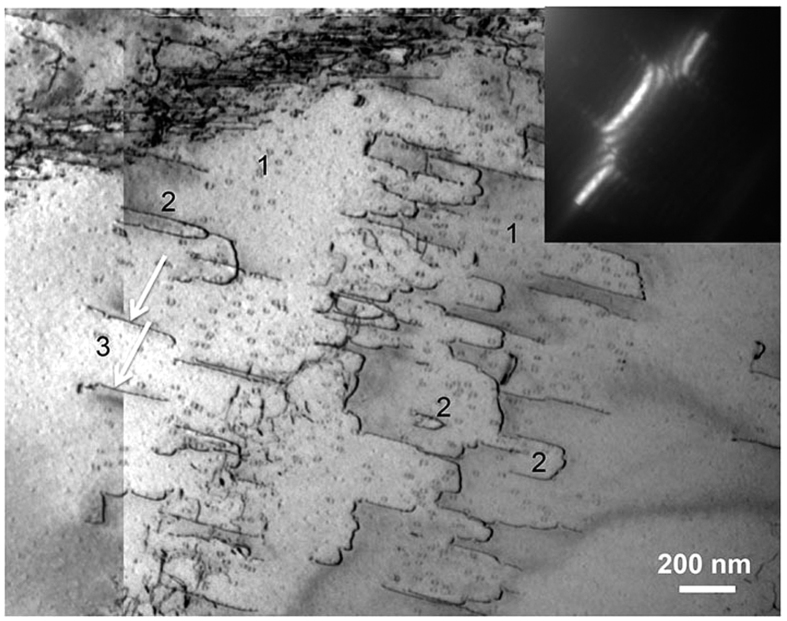
Composition of two bright-field Bragg-contrast images using the (0 −1 −1) reflection at the [1 0 0] zone axis. The numbers label dislocation loops of different expansion (see text). The inset shows a CBED pattern of the dislocation segments labelled “3”.

Figure 5 displays a composition of two Bragg-contrast images at a different specimen location using the (0 −1 −1) reflection at the [1 0 0] zone axis. Two slip bands can be seen, a dense and narrow one in the upper left and a wider, less dense one diagonally in the micrograph. In the latter slip band we find dislocation loops of various sizes. Outside of the slip bands we find hardly any loops (see also e.g. Fig. 2), which shows that they are created during the deformation process and are not artefacts of the specimen-preparation process.

Labelled in the figure are small closed loops of a diameter of about 30 nm (1) similar to those also seen in Fig. 2, larger loops with one visible edge segment assuming an elongated shape (2), and parallel straight segments (3). For the latter, it is not obvious that the two segments belong to one loop, but their screw dipole character can be demonstrated: The inset in Fig. 5 displays a CBED pattern using the (0 −2 −2) Kikuchi line at the [1 0 0] zone axis, which simultaneously crosses both dislocation segments of loop (3). Two fourfold splittings with opposite sign can be seen, which is due to the opposing line directions of the segments (a situation similar to that shown in Fig. 3d, at a wider distance of the segments). The full CBED analysis reveals that the Burgers vector of the loop is a [1 1 1], and tilting analysis shows that this also corresponds to the line direction. This result is in agreement with our previously described analyses – the straight dislocation segments are pure screws and their Burgers vector corresponds to a full body diagonal of the cubic unit cell.

**Figure 6:**
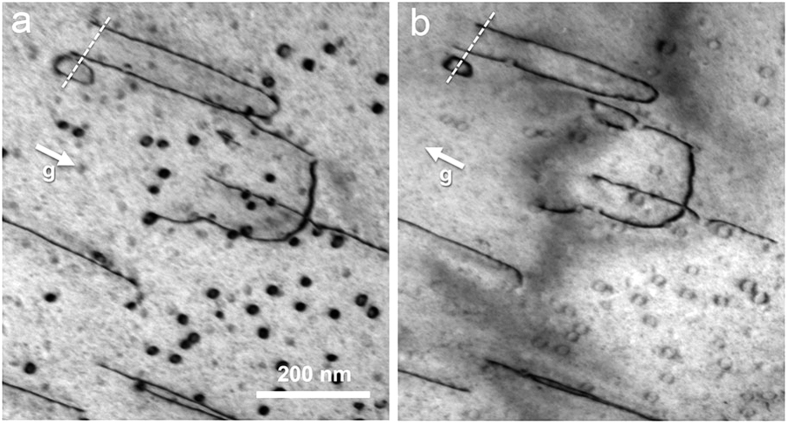
Bright-field Bragg-contrast micrographs of dislocation loops in the deformed Al_28_Co_20_Cr_11_Fe_15_Ni_26_ high-entropy alloy. (**a**) imaged using the (0 1 1) reflection. (**b**) imaged using the (0 −1 −1) reflection close to the [1 0 0] zone axis. The respective directions of **g** are indicated by solid arrows. Along the dashed lines the expansion of the loops was measured.

In order to further investigate the nature of the loops we took image series with opposing imaging vectors (i.e., using reflections ±**g**). Figure 6 displays a portion of the less dense slip band of Fig. 5, containing loops of different expansion. In Fig. 6a, the dislocations are imaged using the reflection (0 1 1), and Fig. 6b using (0 −1 −1) for imaging (both at the [1 0 0] zone axis). The respective directions of **g** are indicated by solid arrows. The smallest loops in Fig. 6a appear as small circles of strong contrast. Imaged with the opposing imaging vector in Fig. 6b, they appear with a weaker contrast of two larger arcs. The connection points of the two arcs lie on a line perpendicular to **g**.

Also for the larger loops, the imaged width varies considerably and hence the apparent segment positions display a relative shift upon changing the sign of **g**. Measured along the dashed line in Fig. 6, the smaller closed loop (lower) has an apparent expansion of 33.7 nm and 22.9 nm imaged using (0 1 1) and (0 −1 −1), respectively. Likewise, the apparent expansion of the larger loop (upper) amounts to 64.0 nm and 51.5 nm. The difference in apparent loop expansion hence amounts to about 12 nm.

**Figure 7:**
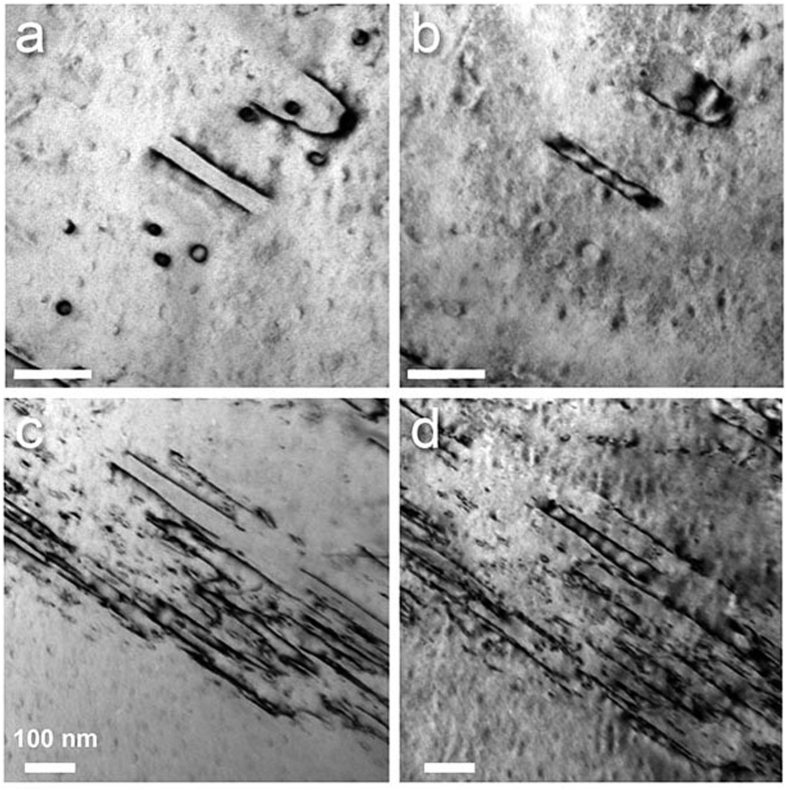
Bright-field Bragg-contrast micrographs of dislocations in the deformed Al_28_Co_20_Cr_11_Fe_15_Ni_26_ high-entropy alloy. (**a**,**c**) imaged using the (1 0 −1) reflection. (**b**,**d**) imaged using the (−1 0 1) reflection at the [1 0 1] zone axis. The scale bar is 100 nm in all images.

Finally, we investigated the faint fringe contrast attached to the straight screw segments, as seen in Figs 2 and 3, under variation of the sign of the imaging vector. Figure 7a,b show Bragg-contrast images of a set of dislocations imaged using the (1 0 −1) and the (−1 0 1) reflection at the [1 0 1] zone axis, respectively. The fringe extends about 10 to 20 nm away from the core region. As described above, the relative positions of the dislocation segments shift upon changing the sign of **g**. Also, fringe contrast under these imaging conditions is located on either side of the dislocation core in Fig. 7a,b. For narrow parallel dislocations the fringe contrast of the individual segments overlaps in Fig. 7b and resembles a stacking-fault contrast. Furthermore it can again be seen that the small loops only show considerable contrast for one sign of the imaging vector, here (1 0 −1). Figure 7c,d show a similar example in a denser slip band.

## Discussion

We have analysed dislocations in a plastically deformed Al_28_Co_20_Cr_11_Fe_15_Ni_26_ HEA. This non-equiatomic HEA consists of a matrix of B2 structure with a small volume fraction of small, spherical inclusions of disordered bcc structure.

The microstructure of the deformed material exhibits distinct slip bands, i.e. the deformation takes place inhomogeneously. Slip bands of different orientation are found, which shows that more than one slip system is activated. Within the slip bands we mainly find straight, parallel dislocation segments. Slip bands imaged in edge-on orientation (e.g. Fig. 2a), reveal that they have a finite width of the order of 1 μm, which indicates that cross slip frequently occurs. This is commonly observed for deformation mechanisms involving screw dislocations in bcc and B2 structures^28^,^29^.

Analysis by CBED and tilting experiments in the TEM reveals that these segments are pure screw dislocations with 〈1 1 1〉 type Burgers vector moving on {1 1 2} type slip planes. The finding of {1 1 2} slip planes indicates that the core of the dislocations may be degenerate and spread onto three intersecting {1 1 0} planes^28^,^30^. We frequently find pairs of straight dislocations with opposing Burgers vector constituting screw dipoles. As a consequence of their dipole character, the screw segments display a distinct shift of their apparent relative position upon change of the sign of **g**^26^.

CBED analysis of dislocations does not only reveal the direction but also the length of their Burgers vector. We find that the dislocations in the Al_28_Co_20_Cr_11_Fe_15_Ni_26_ HEA possess an a 〈1 1 1〉 Burgers vector, which is a full body diagonal of the unit cell and corresponds to a length of 0.50 nm. In terms of the B2 lattice, the dislocations are thus perfect superdislocations.

There are few other studies on dislocations in HEAs available in the literature to compare our results to. Couzinié *et al*.^22^ investigated dislocations in deformed equiatomic TiZrHfNbTa HEAs, which has a bcc structure. They report on screw dislocations of type a/2 〈1 1 1〉 as expected for this structure, and a heterogeneous distribution of the dislocations in bands. Otto *et al*.^21^ analysed dislocations in deformed FeCoCrMnNi. This material is an fcc HEA, and the authors report on slip of a/2 〈1 1 0〉 dislocations on {1 1 1} planes as expected for this structure. Note, however, that in both these studies, dislocations were analysed by contrast extinction, which reveals the direction but not the length of their Burgers vector. It remains unclear, how the authors concluded on the lengths specified. It can be assumed that they were deduced by analogy to similar structures in conventional metals.

There are no previous reports on dislocation analyses in any HEA in the Al-Co-Cr-Fe-Ni alloy system or other HEAs with B2 structure. However, a vast body of literature is available on dislocations in intermetallic B2 compounds. The comparison of our results with these allows for further conclusions.

The deformation of B2 intermetallics is commonly observed to take place by slip of 〈1 1 1〉 dislocations. Depending on the dislocation core structure and the antiphase-boundary (APB) energies, slip takes place on {1 1 0} or {1 1 2} planes^28^,^31^. Typically, in B2 intermetallics and bcc structures a Peierls mechanism controls the motion of screw dislocations^29^. The edge components have a distinctly higher mobility than the screws, leading to strongly elongated dislocation loops with prominent screw segments. As a consequence, in TEM specimens mostly straight screw dislocations are found and segments belonging to the same loop are observed as screw dipoles. These characteristics of typical B2 intermetallics (for a review see ref. 31) compare well to our results in the Al_28_Co_20_Cr_11_Fe_15_Ni_26_ HEA. Slip systems of type {1 1 2}〈1 1 1〉 are e.g. found for low-temperature deformation of Al-Fe^32^, the deformation of β-CuZn at low and elevated deformation temperatures^33^, and NiAl compressed at room temperature along the hard [0 0 1] direction^34^.

Superdislocations of 〈1 1 1〉 type are also found in other B2 intermetallics, e.g. in FeAl, β-CuZn, AgMg, etc.^31^. It is usually observed, however, that the 〈1 1 1〉 superdislocations dissociate into superpartials. The dissociation distance depends on the APB energy and for screws ranges e.g. from 4 to 20 nm in B2 ordered Fe-Al alloys^35^ and from about 5 to 8 nm in β-Cu-Zn^36^. In the present study we determined an upper bound for dislocation dissociation of 1 nm. This means that in the Al_28_Co_20_Cr_11_Fe_15_Ni_26_ HEA, the superdislocations are undissociated or the dissociation width is exceedingly small.

Few cases for undissociated 〈1 1 1〉 superdislocations in intermetallics are known. Veyssière and Noebe^34^ in a weak-beam study resolved no dissociation of 〈1 1 1〉 superdislocations gliding on {1 1 2} planes in NiAl, a situation very similar to that in the present study. These authors concluded on a dissociation width, if any, of 1 to 5 nm and calculated an APB energy larger than 750 mJ/m^2^ and 500 mJ/m^2^ in the {1 1 2} and {1 1 0} planes, respectively. Making the rather weak assumption that there are no other factors determining the dissociation of superdislocations in HEAs, we have to conclude that in Al_28_Co_20_Cr_11_Fe_15_Ni_26_ the APB energies are also very high. This means that the B2 structure has a surprisingly high degree of order. Indeed, recently in the structurally similar system Al_1.5_CoCrCuFeNi, first evidence for the presence of a considerable degree of B2 order was found by Williams *et al*.^37^. These authors demonstrated that one sublattice is preferably occupied by Al, the other preferably by Ni and Co, while Cr and Fe are present on both sublattices. Their results suggest that the matrix of the Al_28_Co_20_Cr_11_Fe_15_Ni_26_ HEA should rather be considered an imperfectly ordered intermetallic than a partially ordered HEA phase.

If the APB energies in the Al_28_Co_20_Cr_11_Fe_15_Ni_26_ HEAs are very high, spreading of the core is inhibited, which in turn implies a low mobility of the superdislocations. Spreading of the core is required to reduce Peierls friction, which, to a crude approximation can be regarded as inversely proportional to the core extension^34^.

We have furthermore observed a fringe contrast attached to the straight dislocation segments, which implies that the elastic strain field of the dislocations extends considerably far from the core. The exact origin of the fringe contrast can only be identified employing accompanying Bragg-contrast image simulations. By now we may hypothesise that the fringe contrast is either a direct consequence of the large Burgers vector of the undissociated superdislocations and/or that local atomic rearrangements take place in the vicinity of the dislocation core region.

However, all these factors, i.e. a high Peierls friction and the extended strain field, decrease the mobility of the dislocations in the Al_28_Co_20_Cr_11_Fe_15_Ni_26_ HEA. Our results thus provide a possible explanation for the high strength of these materials as a direct consequence of their dislocation structure. Note that recently Zhang *et al*. reported a similar conclusion, identifying undissociated a/2 〈1 1 0〉 type dislocations as a major source of strength in single phase fcc FeCoCrMnNi HEAs^38^.

The structure of the presently investigated material also constitutes that of equiatomic AlCoCrFeNi^25^ and AlCoCrCuFeNi^24^, and of other HEAs in the Al-rich region of these systems^39^,^40^. These HEAs consist of a matrix of B2 structure and a second disordered bcc phase. The volume ratio of the B2 matrix and the disordered bcc phase depend on the composition and increases with the Al content of the composition. In the Al-Co-Cr-Cu-Fe-Ni system, segregated Cu adds as a third phase. Our conclusions based on results on the B2-ordered Al_28_Co_20_Cr_11_Fe_15_Ni_26_ alloy are therefore expected to be valid for these B2-based materials as well, which indeed show very high strength^13^,^16^,^19^. Our results may also be relevant for the understanding of the deformation mechanism in equiatomic HEAs in these systems with additional doping by Ti, Mn or V^41^,^42^ or Mo^43^.

In the slip bands we also find dislocation loops of different size. The smallest loops occur at a high density and have diameters of about 30 nm. They display characteristic contrast behaviour upon variation of the sign of the imaging vector, strongly resembling the “inside-outside” contrast as described e.g. by Edington^44^. The small loops can either be prismatic loops created during cross slip of the edge dislocations or Orowan loops formed by the overcoming of obstacles in the slip plane by moving dislocations. We observe (Figs 2,5,6 and 7) that the sign of all loops in a given slip band is always identical and that the diameter of the loops is always very similar. These observations strongly indicate that the present small loops are Orowan loops created during the movement of dislocations of the dominant Burgers vector direction within a slip band. Prismatic loops created by cross slip are expected to form with both signs at approximately equal occurrence and with a much wider size distribution. This conclusion is congruent with our STEM observations under simultaneous use of a HAADF- and a bright-field detector, revealing that the small loops are always located around small spherical inclusions (see Supplementary information Fig. S4). Details of this study exceed the scope of the present paper and will be presented in a forthcoming publication.

## Additional Information

**How to cite this article**: Feuerbacher, M. Dislocations and deformation microstructure in a B2-ordered Al_28_Co_20_Cr_11_Fe_15_Ni_26_ high-entropy alloy. *Sci. Rep.* **6**, 29700; doi: 10.1038/srep29700 (2016).

## Supplementary Material

Supplementary Information
