# Supplementary material for: Dislocations and deformation microstructure in a B2-ordered Al28Co20Cr11Fe15Ni26 high-entropy alloy
Source: Sci Rep. 2016 Jul 19;6:29700. doi: 10.1038/srep29700 (PMC4949441; doi:10.1038/srep29700)
Supplement: Supplementary Information [file srep29700-s1.pdf]

Supplementary Information

Dislocations and deformation microstructure in a B2-ordered $Al_{28}Co_{20}Cr_{11}Fe_{15}Ni_{26}$ high-entropy alloy

Michael Feuerbacher
Peter Grünberg Institut PGI-5, Forschungszentrum Jülich GmbH, D-52425 Jülich, Germany

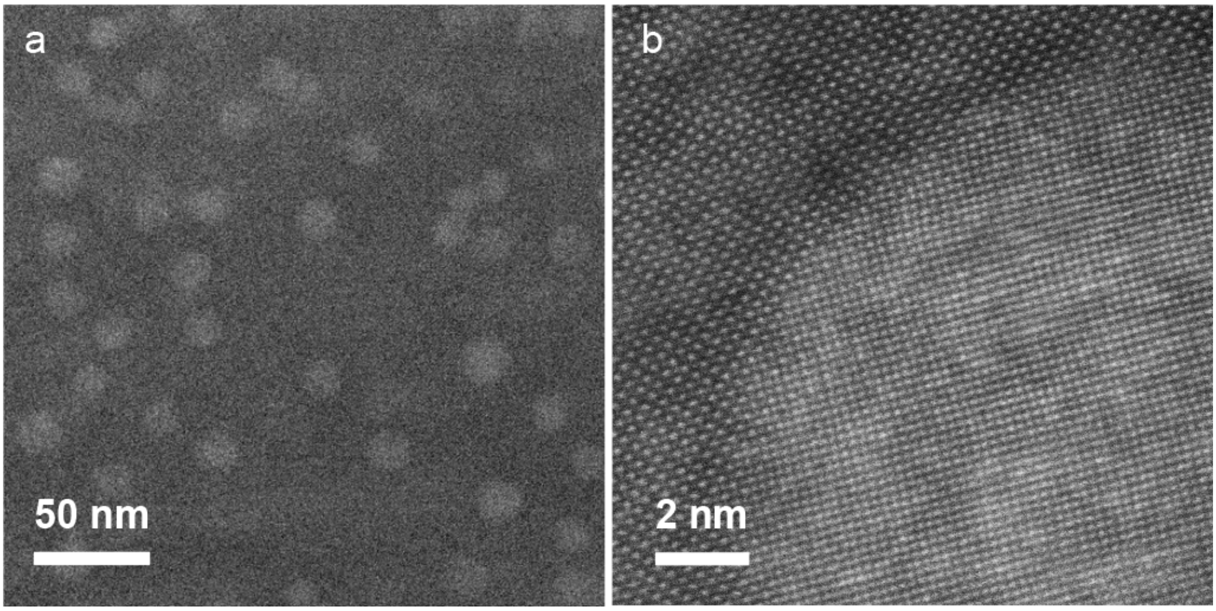

Figure S1: STEM micrographs of spherical inclusions in the $Al_{28}Co_{20}Cr_{11}Fe_{15}Ni_{26}$ high-entropy alloy. (a) Low magnification demonstrating the monodispersity of the inclusions. (b) High magnification along [1 0 0]. The spherical inclusion in the lower right corner has a disordered bcc structure resulting in equal intensity on all atomic column positions. In contrast, the matrix displays lower column intensity on one of the B2 sublattices. In all directions the lattice planes of the inclusion are perfectly congruent with those of the matrix.

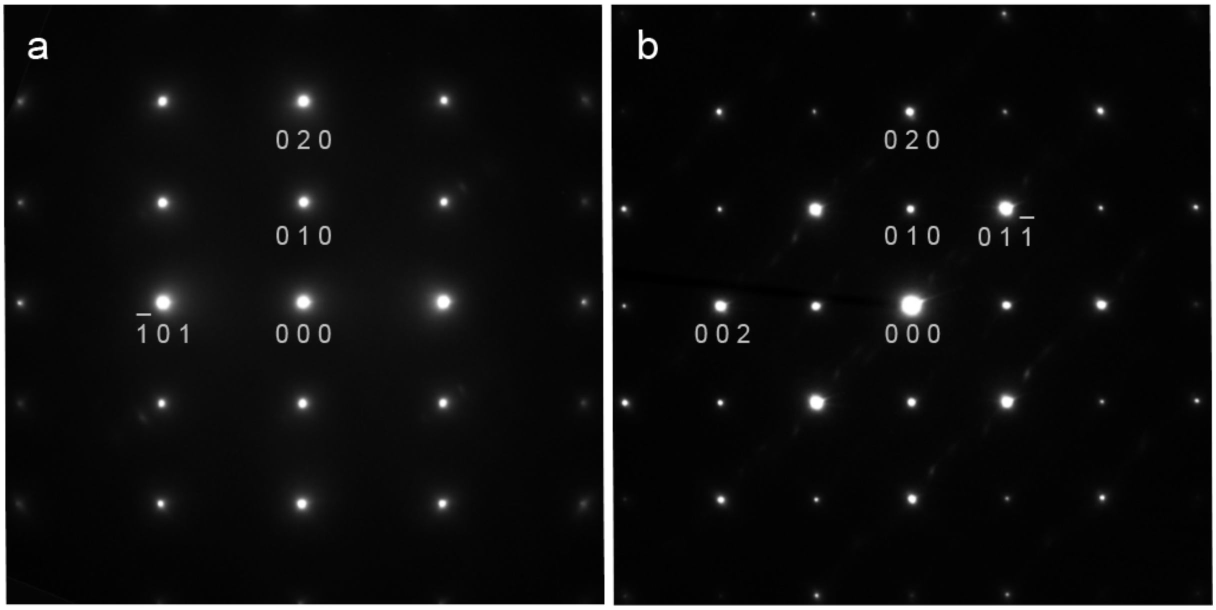

Figure S2: Electron diffraction patterns of the $Al_{28}Co_{20}Cr_{11}Fe_{15}Ni_{26}$ high-entropy alloy. (a) Along the [1 0 1] zone axis. (b) Along the [1 0 0] zone axis. Both diffraction patterns contain {0 1 0} type superstructure reflections forbidden in bcc, which demonstrates the presence of B2 order. The diffraction patterns are typical examples of several patterns taken at various positions in different samples. The selected area of the specimen contributing to the diffraction patterns is about 300 μm^2.

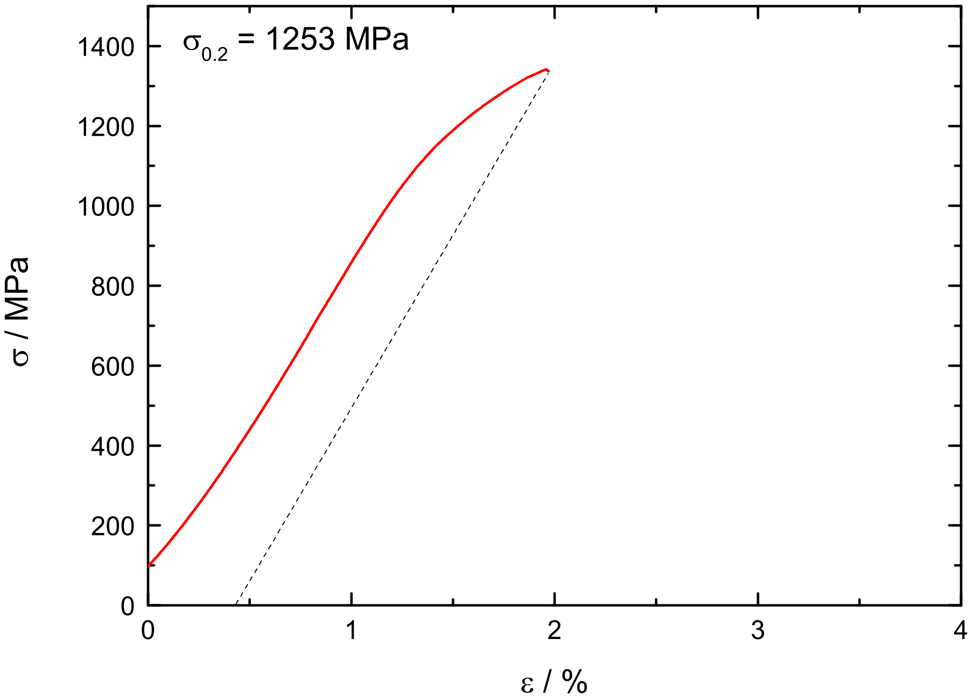

Figure S3: True stress – true strain curve of the $Al_{28}Co_{20}Cr_{11}Fe_{15}Ni_{26}$ high-entropy alloy at room temperature and a strain rate of 10^{-4} s^{-1}. The dotted line is parallel to the linear elastic part of the curve extrapolated through σ(0 %) = 0 MPa and demonstrates that the sample was deformed plastically by a strain of slightly less than 0.5 % before unloading.

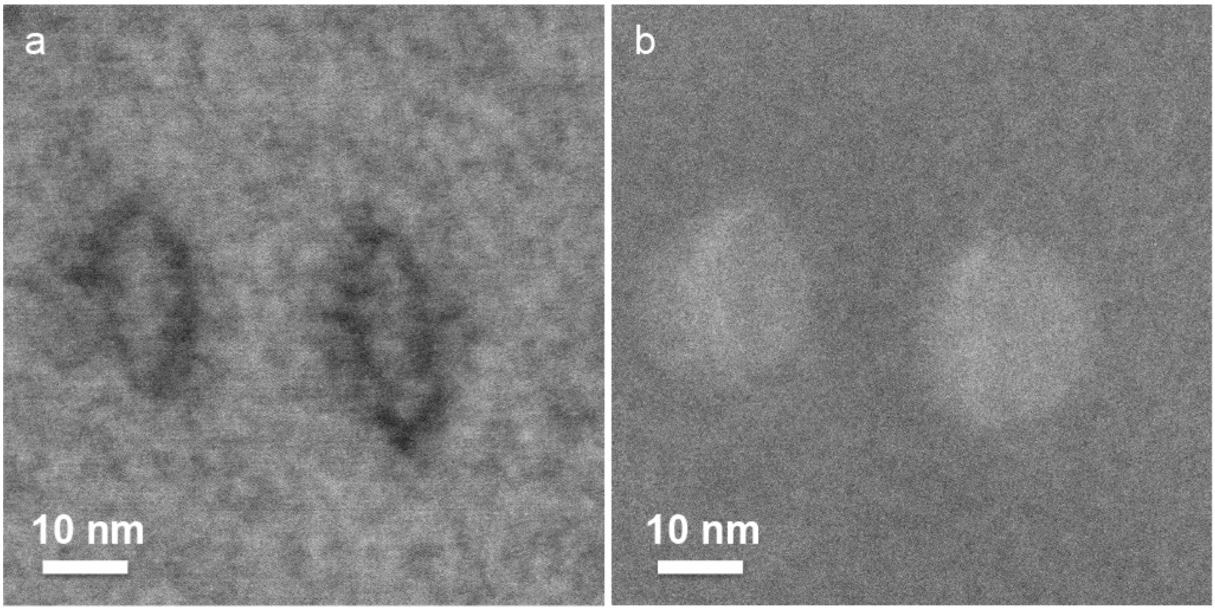

Figure S4: STEM micrographs of the $Al_{28}Co_{20}Cr_{11}Fe_{15}Ni_{26}$ high-entropy alloy. (a) Using a bright-field detector the strain-field contrast of two small dislocation loops is seen. (b) The same specimen region taken with a high-angle annular dark field detector, which images contrast due to the presence of different atomic species. Two spherical bcc inclusions can be seen. The combination of both images reveals that the small loops are located around the spherical inclusions, which indicates that they are created by an Orowan mechanism.
